# New Conclusions Regarding Comparison of Sevelamer and Calcium-Based Phosphate Binders in Coronary-Artery Calcification for Dialysis Patients: A Meta-Analysis of Randomized Controlled Trials

**DOI:** 10.1371/journal.pone.0133938

**Published:** 2015-07-31

**Authors:** Caixia Wang, Xun Liu, Yongming Zhou, Shaomin Li, Yanbing Chen, Yanni Wang, Tanqi Lou

**Affiliations:** 1 Department of Nephrology, the Third Affiliated Hospital of Sun Yat-sen University, Guangzhou, China; 2 Department of Nephrology, The Affiliated Tianyou Hospital, Wuhan University of Science and Technology, Wuhan, China; 3 Medical Genetics Center, Guangdong Women and Children Hospital, Guangzhou, China; University of Bologna, ITALY

## Abstract

**Background:**

Sevelamer hydrochloride is used widely, but its impact upon cardiovascular calcification, cardiovascular mortality, all-cause mortality and hospitalization is not known.

**Outcomes:**

Primary outcome was cardiovascular calcification (coronary artery calcification scores (CACS) and aortic calcification scores (ACS)). Secondary outcomes were serum characteristics, hospitalization, cardiovascular mortality and all-cause mortality. Risk ratio (RR), mean differences and standard mean difference with 95% confidence intervals (CIs) were pooled using random- or fixed-effects models.

**Results:**

We identified 31 studies (on 23 randomized controlled trials with 4395 participants). An analysis pooling showed a significant decrease in serum levels of phosphate with calcium-based phosphate binders (CBPBs) by 0.17 mg/dL [mean difference (MD), 95% CI, 0.03, 0.31] than sevelamer. A significant difference in the change of CACS by –102.66 [MD: 95% CI, –159.51, –45.80] and ACS by –1008.73 [MD, 95% CI, –1664.75, –352.72] between sevelamer and CBPBs was observed. Prevalence of hypercalcemia (serum levels of calcium >10.2–10.5 mg/dL and >11.0 mg/dL) was significantly smaller for sevelamer (RR = 0.44, 95% CI, 0.33, 0.58; RR = 0.24, 95% CI, 0.14, 0.40). No significant difference was found in hospitalization, all-cause mortality or cardiovascular mortality.

**Conclusions:**

This meta-analysis suggests that sevelamer benefits dialysis patients in terms of CACS, ACS and hypercalcemia.

## Introduction

Chronic kidney disease (CKD) is a major public-health problem [1]. As a major therapy for patients with end-stage renal disease (ESRD), renal replacement therapy is used widely all over the world. However, dialysis patients can suffer from mineral metabolism. Also, cardiovascular disease is the most common cause of death, accounting for more than one-half of cases [2–4].

Higher levels of phosphate in serum are associated with worse outcomes in dialysis patients, so different types of therapies have been employed to deal with this problem. Phosphate binders taken with meals, which bind dietary phosphate, play an important part in the treatment of hyperphosphatemia [5]. Dietary phosphate binders are used widely. Calcium-based agents were traditionally employed as first-line therapy [6] but their use can result in hypercalcemia and high levels of calcium-phosphate products, which are associated with cardiovascular mortality and mortality in ESRD. Hence, magnesium- and aluminum-based agents have started to be used.

New non-calcium, non-magnesium, and aluminum-free phosphate-binding means agents such as sevelamer have been reported to reduce the Medicare costs of inpatients compared with calcium binders [7] without alerting serum levels of calcium. As a type of calcium-free agent, sevelamer may has less influence upon serum levels of calcium [8]. However, its impact upon cardiovascular calcification, cardiovascular mortality, all-cause mortality and hospitalization is not known.

Several reviews [9–13] have focused on sevelamer, one of which was conducted in 2010 involving 14 trials and 3271 patients [9]. In that meta-analysis, the authors included predialysis patients and evaluated the level of cardiovascular calcification using coronary artery calcification scores (CACS), graded by computed tomography (CT) and representing the progression or regression of coronary artery disease, [14, 15] in four randomized controlled trials (RCTs) in hemodialysis patients. Jamal et al. (2009) [10] also analyzed cardiovascular calcification by CACS, but found no significant differences in CACS between patient groups and controls. Three of those reviews [11–13] considered biochemical outcomes, and one review also evaluated the effect of sevelamer upon all-cause mortality, cardiovascular events, and other adverse events [11]. Since then, several trials related to this issue have been published. It seems that an updated review of the evidence would be of great use to clinicians and decision-makers. Hence, we conducted a meta-analysis of published RCTs on the effectiveness and safety of sevelamer in dialysis patients.

## Materials and Methods

### Data Sources and Literature Searches

We undertook a systematic meta-analysis of RCTs according to Preferred Reporting Items for Systematic Reviews and Meta-analyses (PRISMA) guidelines (S1 Fig) [16]. We conducted a MEDLINE literature search to identify all relevant studies using the search terms ‘sevelamer hydrochloride’, ‘sevelamer’, or ‘RenaGel’ from January 1998 to November 2013 and searched PUBMED, EMBASE (‘sevelamer hydrochloride’/exp OR ‘sevelamer hydrochloride’ OR ‘sevelamer’/exp OR sevelamer AND (‘renagel’/exp OR renagel) 1811), the specialized register of the Cochrane Renal Group, and the Cochrane Central Register of Controlled Trials to identify all RCTs studying the effects of sevelamer hydrochloride using similar search terms.

We also searched (manually) the abstracts of conference proceedings of the American Society of Nephrology from 1998 to 2013. However, we did not have access to RCTs that were not reported.

**Fig 1:**
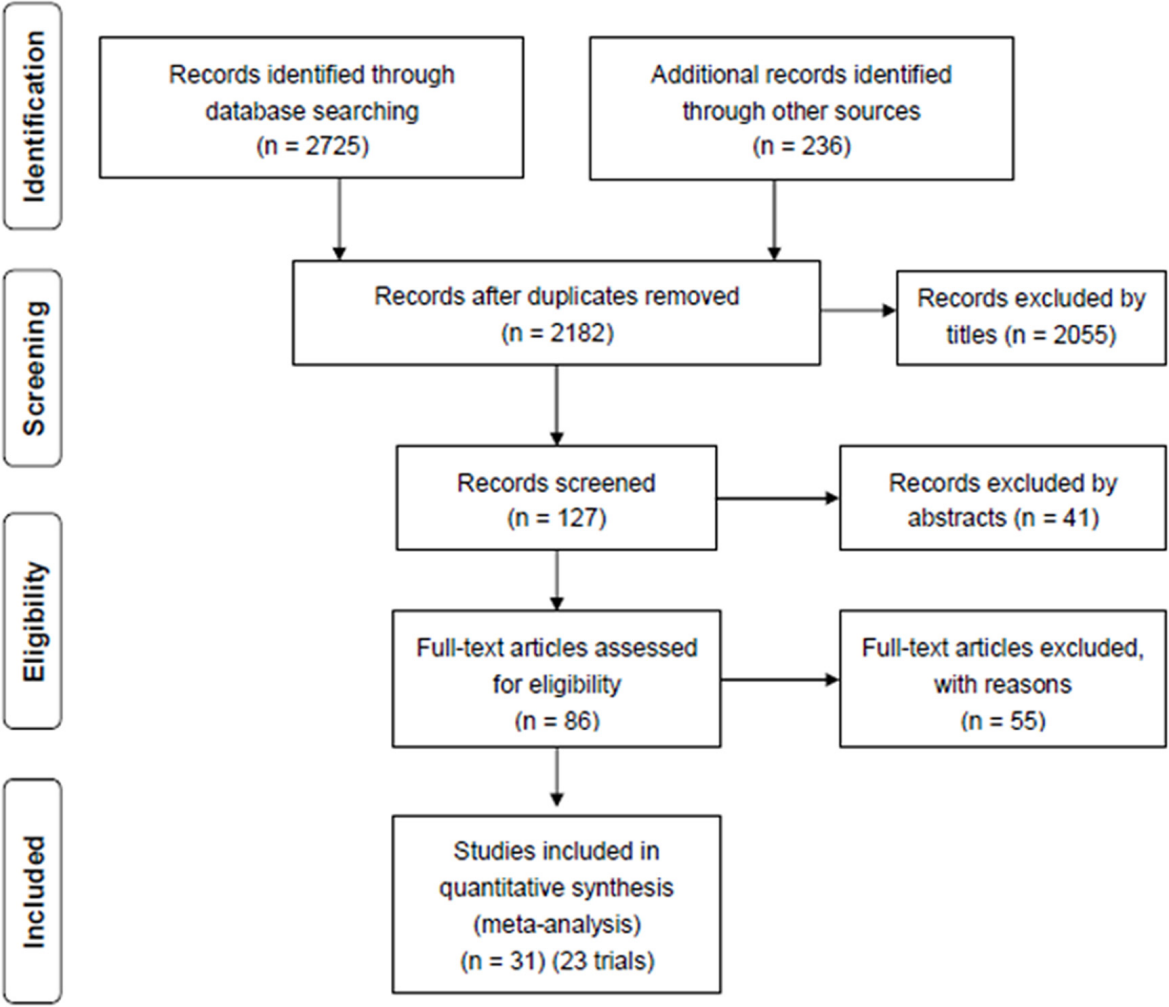
Flow diagram of studies considered for inclusion.

Restrictions on language or dates were not imposed in our searches. Finally, we found 2961 studies for the analysis. After screening, 31 studies (on 23 trials) were included (Fig 1) in the analysis.

### Study Selection

All RCTs that studied dialysis ESRD adults (age ≥18 years) and compared sevelamer to any calcium-based phosphate binder (CBPB) were included. Included studies are assumed to have analyzed the effect of phosphate binders on serum levels of phosphate or calcification of coronary arteries. Studies comparing sevelamer to any other types of phosphate binders or no phosphate binders were excluded. Titles and abstracts were reviewed by two reviewers independently, as well as the full-text articles.

### Data Extraction and Quality Assessment

Data were extracted by two authors. A third reviewer checked the extracted data for accuracy. The following data were extracted: country of origin; year of publication; sample size; study design; mean age; percentage of men; mean duration of dialysis; prevalence of diabetes. The quality of trials was assessed by Review Manager 5.2 (Oxford, UK) according to the *Cochrane Handbook for Systematic Reviews of Interventions* (S2 Fig and S3 Fig) [17]. Levels of evidence were evaluated by the GRADE profiler (S4 Fig) [17]. A third person was available if there was disagreement concerning extraction and/or assessment of the quality of data.

### Synthesis and Analysis of Data

We undertook meta-analyses using Review Manager 5.2 and meta-regression by comprehensive meta-analysis (CMA). Mean difference (MD) and standard mean difference (SMD) were used to pool results for continuous outcomes (e.g. serum levels of phosphate and calcium), and we also computed pooled risk ratios (RRs) for dichotomous outcomes (e.g. cardiovascular mortality, all-cause mortality). We used change-from-baseline results rather than final values in the analysis of CACS and aortic calcification scores (ACS) to evaluate the effect of phosphate binders upon vascular calcification. Pooling methods that account for the with-inpatient-correlation from crossover trials were used to combine data from crossover and parallel continuous trials [18]. A fixed- (used if I^2^≤25%) and a random-effects model (used if I^2^≥50%) was used to analyze data.

Ninety-five percent confidence intervals (95% CIs) were provided for all pooled estimates. Heterogeneity was assessed using the Cochrane Q test. I^2^ index (which describes the percentage of total variation across studies due to true heterogeneity rather than chance) and P values were also used. Publication bias was assessed using Funnel plots.

## Results

### Selection and Characteristics of Studies

A total of 2961 potentially relevant citations were identified and screened. Eighty-six articles were retrieved for detailed evaluation, of which 31 (23 trials were analyzed in total) fulfilled the eligibility criteria (Fig 1). Detailed characteristics and a summary of all 31 studies (23 trials) are displayed in Tables 1 and 2. Multiple publications with no unique result were excluded from screened studies. However, unique results were extracted and studies (as well as abstracts) containing unique results were also displayed. Block 2007 [19], was a follow-up analysis of earlier studies [20–21] that compared sevelamer with CBPBs. The study of Barreto 2005 [22] was a published abstract of the study of Barreto 2008 [23], and contained some data that the full report did not mention or did not describe in detail. Chertow 2003 [24] is a short term follow-up trial which evaluated the same patients investigated in Asmus 2005 [25] which was a long term follow-up trial for them. Chertow 2002 [26], Raggi 2004 [27] and Ferramosca 2005 [28] et. al also shared data from the same patients. However, all of them (containing the same cohort of participants) were extracted only once. Sample size of studies varied from 13 patients to 2103 patients (a total of 4395 participants). Mean age was 57.9 years. Duration of dialysis was from 3 months to 18 years. Prevalence of diabetes ranged from 0% to 60%.

**Table 1:** Detailed characteristics of the studies.

Study	Country	Modality^a^	Duration^b^ (yr)	Follow-up^c^ (wk)	S dose^d^ (g/d)	CBPB dose (g/d)	Sample size
**Asmus 2005**	USA	HD	5.1	104	6.9	4.3	72
**Barreto 2005**	Brazil	HD	NR	52	NR	NR	101
**Barreto 2008**	Brazil	HD	3.1	52	12	2.028	101
**Bleyer 1999**	USA	HD	NR	10	NR	NR	84
**Block 2005**	US	HD	0.25	78	8	2.3	129
**Block 2007**	USA	HD	NR	189	NR	NR	127
**Braun 2004**	Europe	HD	5.3	52	5.9	3.9	114
**Cancela 2011**	Brazil	HD	3.1	52	NR	NR	72
**Chertow 1999**	USA	HD	NR	16	NR	0.9	71
**Chertow 2002**	US, Ger, Au^e^	HD	3.3	52	6.5	4.6	200
**Chertow 2003**	USA	HD	2.5	52	2.4	2	108
**Evenepoel 2009**	NR	PD	1.2	12	4.8	4.8	143
**Ferreira 2008**	USA	HD	＞3.5	55	5.0	4.0	119
**Francisco 2010**	Europe	HD	5.0	26	3.2	1.74	255
**Ferramosca 2005**	USA	HD	4.8	53	6.5	4.3	108
**Gallieni 2005**	NR	HD	NR	12	0.403	0.403	114
**Garg 2005**	US, Ger, Au^e^	HD	3.3	52	NR	6.5	200
**Herva’s 2003**	Spain	HD	4.7	34	4.09	3.9	51
**Kakuta 2011**	USA	HD	9.33	52	9	10.5	183
**Lin 2010**	Taiwan	HD	3.6	10	2.4	2.0	52
**Liu 2006**	USA	HD	7.3	8	0.4	0.667	70
**Oliveira 2007**	Brazil	HD	NR	54	NR	NR	19
**Peter 2008**	NR	HD	NR	104	NR	NR	2103
**Qunibi 2004**	USA	HD	4.3	8	6.9	7.1	100
**Qunibi 2008**	USA	HD	1.9	52	7.3	5.5	203
**Raggi 2004**	US, Ger, Au^e^	HD	3.3	52	NR	NR	200
**Raggi 2005**	US, Ger, Au^e^	HD	3.3	52	NR	NR	111
**Sadek 2003**	Europe	HD	NR	21	NR	4.85	42
**Shaheen 2004**	SA^f^	HD	3.4	20	2.4	1.8	20
**Suki 2008**	US	HD	3.2	193	6.9	5.3	2103
**Takei 2008**	Japan	HD	12	28	NR	NR	42

Chertow et al. 2003 and Ferramosca et al. 2005 shared the same patients, and Asmus et al. 2005 had a longer follow-up of the two studies. Block et al. 2007 had a longer follow-up than Block et al. 2007. Suki et al. 2008 and Peter et al. 2008 analyzed the same trial. Chertow et al. 2002, Raggi et al. 2004, Raggi et al. 2005 and Garge et al. 2005 analyzed the same trial. Barreto et al. 2005 is an abstract of Barreto et al. 2008, but with different types of data. Abbreviations: HD, hemodialysis; NR, not reported.

^a^Dialysis.

^b^Mean duration of dialysis

^c^Follow-up of trials

^d^Mean dose of sevelamer

^e^USA, German, Austria

^f^Saudi Arabia

**Table 2:** Summary of the studies analyzed.

**No. of studies** ^a^	31
**No. of trials** ^b^	23
**Sample size of the trials** ^b^	4395
**Percentage of female participants** ^b^	41
**Age of participants (yr)** ^b^	57.9
**Percentage of participants with diabetes** ^b^	45
**Body mass index (kg/m2)** ^b^	26.8
**Percent of current smoking (%)** ^b^	15.6
**Cause of ESRD** ^b^ **:**	
** Hypertension (%)**	26.5
** Diabetes mellitus (%)**	41.6
** Glomerulonephritis (%)**	14.1
** Polycystic kidney (%)**	4.0
** Other (%)**	13.8

^a^ All studies having been analyzed are included

^**b**^ The same data were extracted for only once

A total of 31 studies, including an abstract [29] and five posters [22, 30–32], were eligible for the analysis. Those studies compared sevelamer with calcium acetate, calcium carbonate, or both. One study had no baseline washout period. One study included only patients who initiated dialysis recently, and another study included only those on incident hemodialysis. All of the trials were accrued on hemodialysis patients except one, which focused on patients undergoing peritoneal dialysis [33].

### Effect of Sevelamer vs. CBPBs on Serum Measurements

**Fig 2:**
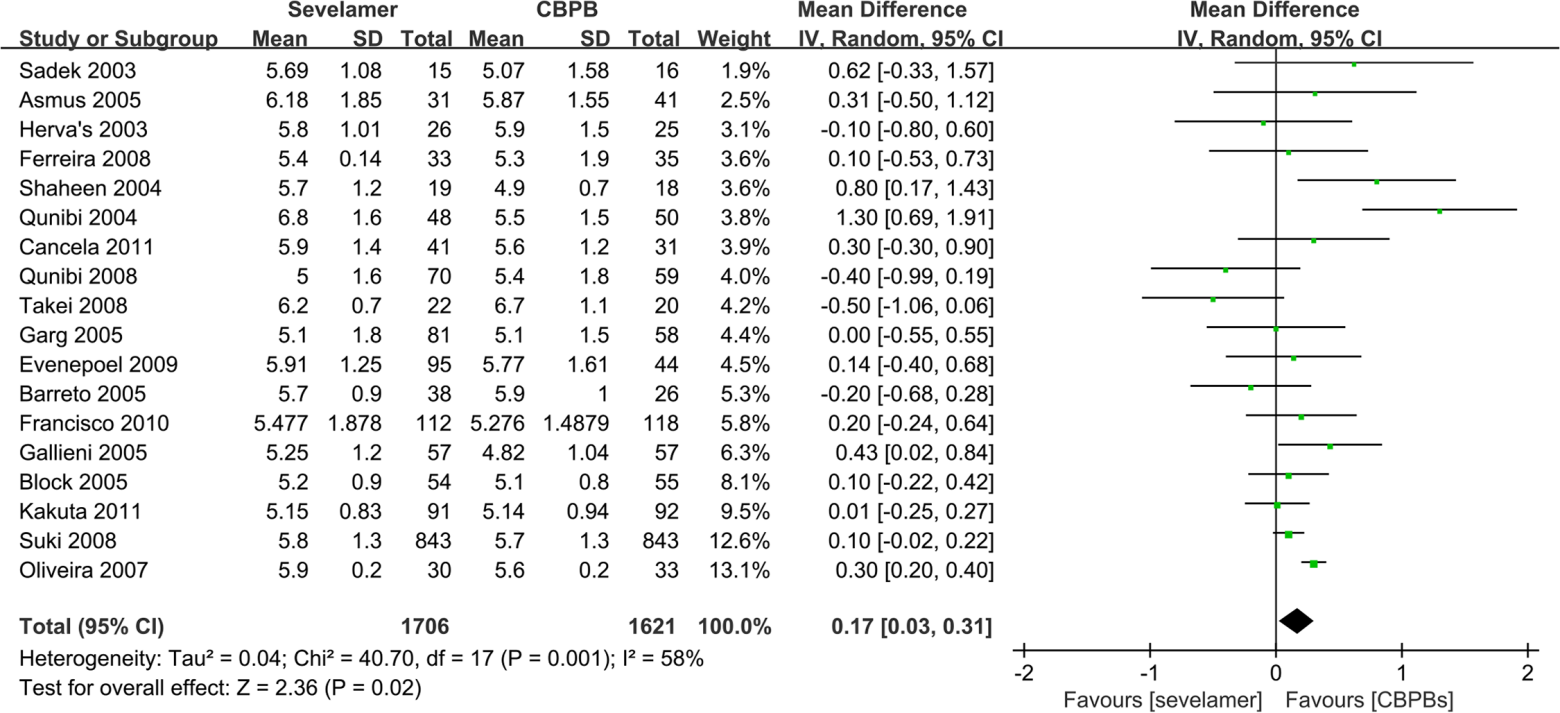
Forest plot of the values of phosphorus.

In an analysis of 18 studies with 3327 participants reporting on serum levels of phosphate (duration of follow-up ranged from 8 weeks to 45 months), a significant decrease in serum levels of phosphate with CBPBs by 0.17 mg/dL (MD, 95% CI, 0.03, 0.31) was observed (Fig 2). All RCTs showed that CBPBs were better than sevelamer for the control of serum levels of phosphate.

Compared with CBPBs, the MD in serum levels of calcium (18 studies; 3425 participants; duration, 8 weeks to 45 months) and in calcium-phosphate product (14 RCTs; 3050 participants; duration, 8 weeks to 45 months) were significantly lower in patients administered sevelamer by –0.24 (95% CI, –0.34, –0.14) and by –0.14 (95% CI, –1.38, 1.10) separately.

### Effect of Sevelamer vs. CBPBs upon Hypercalcemia

**Fig 3:**
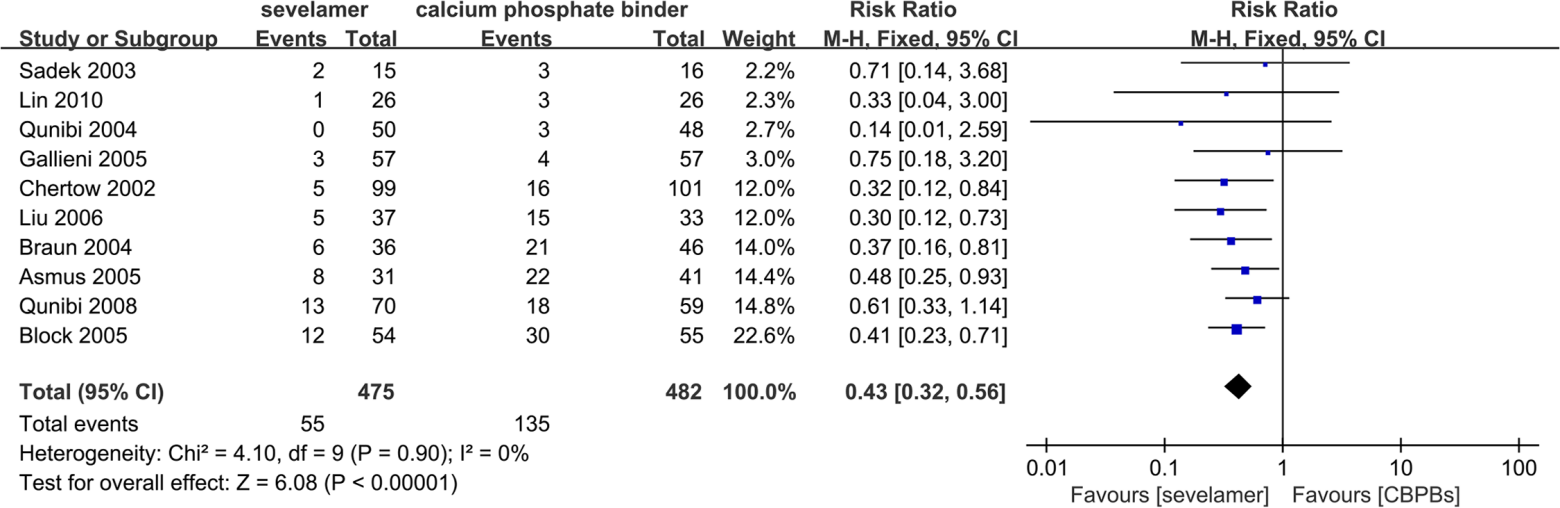
Forest plot of the values of hepercalcemia (above 10.2 mg-dL).

**Fig 4:**
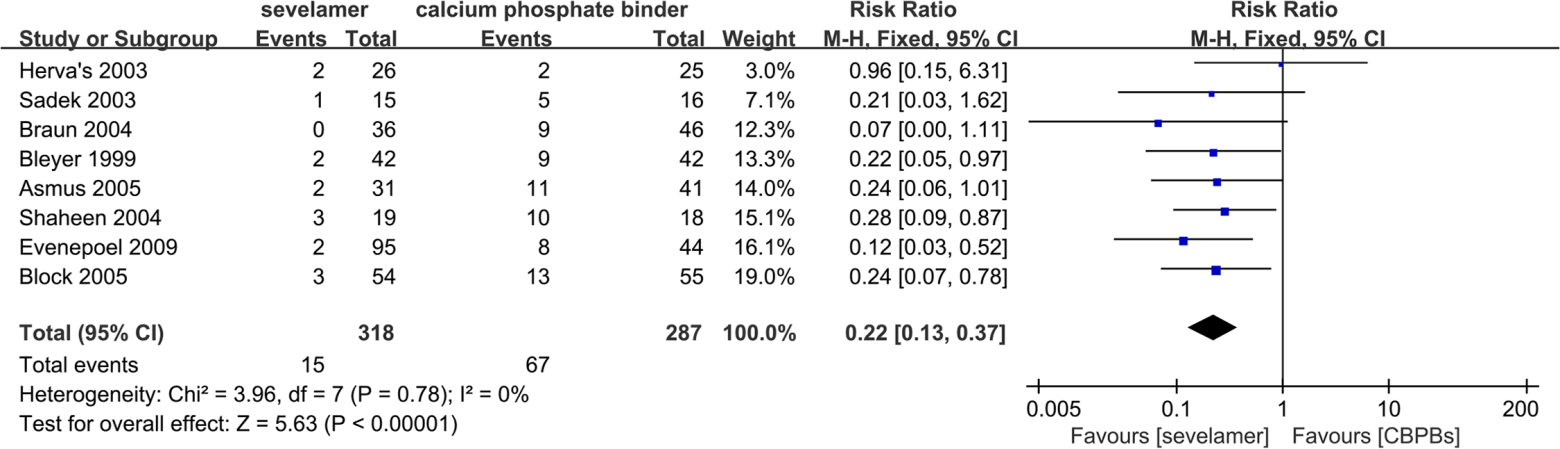
Forest plot of the values of hepercalcemia (above 11.0 mg-dL).

Level of hypercalcemia (defined in all trials as serum levels of calcium >10.2–10.5 mg/dL) reported in ten trials with 957 participants was smaller for sevelamer (RR, 0.43; 95% CI, 0.32, 0.56) compared with CBPBs (Fig 3). When hypercalcemia was defined as serum levels of calcium >11.0 mg/dL (which is viewed as “severe hypercalcemia”), the RR reported by eight trials with 605 patients was 0.22 (95% CI, 0.13, 0.37) (Fig 4). However, no trial reported on the clinical consequences or median duration of hypercalcemia.

### Effect of Sevelamer vs. CBPBs on CACS and ACS

**Fig 5:**
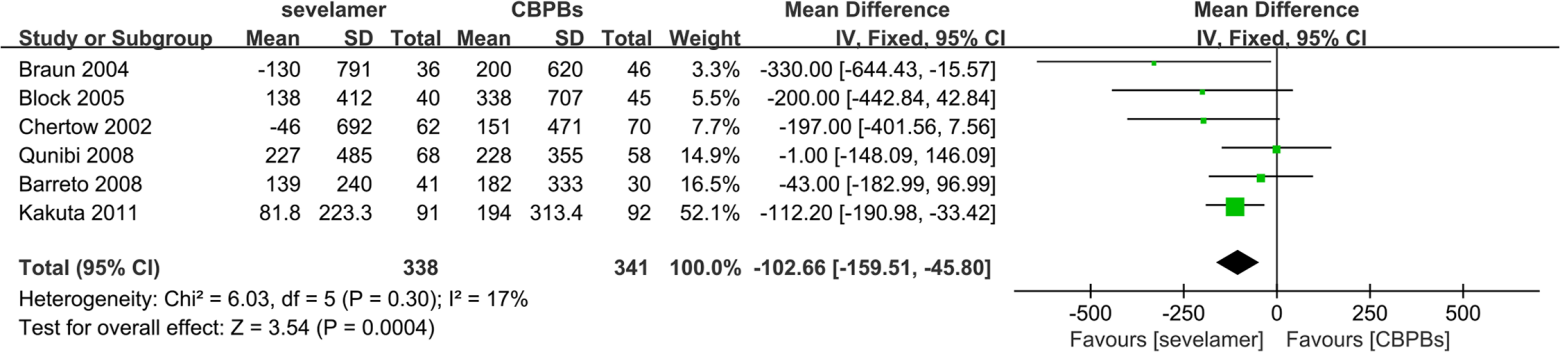
Forest plot of sevelamer vs. calcium phosphate binders on CACS change.

**Fig 6:**
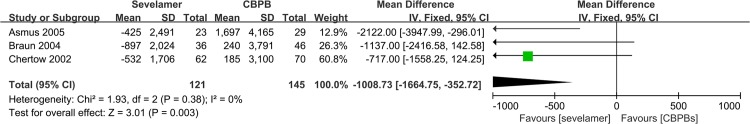
Forest plot of sevelamer vs. calcium phosphate binders on ACS change.

Seven studies with 731 participants, one of which had a sample size of only 52 participants, reported on the change of CACS. Considering the quality of the RCTs, we only included the six trials with 679 patients. The duration of follow-up varied from 26 weeks to 104 weeks. MD was significant, and was lower with sevelamer therapy by –102.66 (MD: 95% CI, –159.51, –45.80) (Fig 5). All RCTs analyzed showed that sevelamer was better for preventing calcification of coronary arteries than CBPB. The change in ACS was also extracted from three studies with 266 patients. Similar to the analysis of CACS, the analysis of ACS showed a significant decrease by –1008.26 (SMD: 95% CI, –1664.75, –352.72) (Fig 6).

Considering that the use of statins (detailed in Table 3) may have an impact on the change of CACS, we performed a linear regression on the change of CACS and the levels of low density lipoprotein (LDL) regulated mostly by statins. We found no significant relationship between CACS and LDL (Beta = -0.013; P = 0.971) (S5 Fig), which indicates that the use of statins has no significant impact on the change of CACS.

**Table 3:** Details of the use of statins.

Study	Statins	Kinds	Details	Evaluation on statins
**Qunibi 2008**	Yes	Atorvastatin	Different statins given time^b^	No definitive conclusions^e^
**Kakuta 2011**	Yes	NR^a^	Different proportion of patients given statins^c^	No significant difference
**Block 2005**	NR^a^	NR^a^	NR^a^	NR^a^
**Barreto 2008**	NR^a^	NR^a^	NR^a^	NR^a^
**Chertow 2002**	NR^a^	NR^a^	NR^a^	NR^a^
**Braun 2004**	Yes	NR^a^	Different proportion of patients given statins^d^	No significant difference^f^

^a^Not reported

^b^Statins were given to calcium group at start, to sevelamer group at week 8 only if their LDL-C levels were not less than 70 mg/dL

^c^8% patients were given statins in sevelamer group, while 11% in cacium group

^d^26% patients were given statins in sevelamer group, while 33% in cacium group

^e^Definitive conclusions about the role of LDL-C lowering in the progression of CAC was unavailable

^f^Statin use was not associated with less progression of coronary artery or aortic calcification in sevelamer or calcium carbonate patients

### Effect of Sevelamer vs. CBPBs on Hospitalizations

Three RCTs with 2348 participants reported on the number of patients hospitalized during the study. The RR was smaller by 0.78 (95% CI, 0.61, 0.99), showing that sevelamer benefited patients with regard to hospitalization. Only one trial [34] reported on the number of days of hospitalization. Sevelamer-treated patients were hospitalized for fewer days (sevelamer (mean), 14.8±27.9; median, 5.0 hospital days/patient-year; calcium (mean), 17.4±32.0; median, 5.8 hospital days/patient-year; P = 0.09) in the trial of Suki et al. 2008 [34] but the difference was not significant.

### Effect of Sevelamer vs. CBPBs on Mortality

Nine trials with 3000 participants reported all-cause mortality, and the duration of follow-up ranged from 20 weeks to 45 months. Three RCTs analyzed all-cause mortality as the primary outcome. The RR was 0.91 (95% CI, 0.79, 1.04) between sevelamer and CBPBs. Three RCTs with 2102 participants reported on cardiovascular mortality, and the RR was also non-significant by 0.94 (95% CI, 0.76, 1.16). As a result, no significant difference was found in all-cause mortality and cardiovascular mortality.

### Heterogeneity and Publication Bias

All data (detailed in Table 4) were analyzed by fixed-effects (I^2^≤50%) and random-effects (I^2^>50%) models. Between-study heterogeneity ranged from 0% to 75%. Among all the data analyzed, no between-study heterogeneity (0%) was observed in the analysis of cardiovascular mortality, all-cause mortality, change in ACS, hospitalization, and hypercalcemia. Between-study heterogeneity was low (I^2^≤25%) in the analysis of change in CACS, and was moderate (25%<I^2^≤50%) in the analysis of serum calcium-phosphate product. Between-study heterogeneity of the data analyzed was high (>50%) for serum levels of phosphate and calcium. We could not undertake a subgroup analysis, so we used the random-effect model to analyze the data: serum levels of phosphorus had an I^2^ = 58% and serum levels of calcium had an I^2^ = 75%.

**Table 4:** Overall outcome summaries.

Outcomes	Studies	Quality^a^	Patients	Overall summary	*I* ^*2*^ & *p*	F^e^ (wk)	Reference
**Sevelamer vs. calcium**							
**Serum phosphate (mg/dL)**	18	High	3327	MD R^b^ 0.17 [0.03, 0.31]	58%; 0.001	49	[20, 22, 24–26, 31–46]
**Serum calcium (mg/dL)**	18	Moderate	3425	MD R -0.24 [-0.34, -0.14]	77%; 0.001	52	[20, 22, 24–26, 31–38, 40–46]
**Serum c×p product** ^d^ **(mg** ^**2**^ **/dL** ^**2**^ **)**	14	Moderate	3050	MD R -0.14 [-1.38, 1.10]	30%; 0.14	50	[20, 24–26, 31, 33, 34, 39–45]
**Change in CACS**	6	High	679	MD F^c^ -102.66 [-159.51, -45.80]	17%; 0.3	62	[20, 23, 25, 26, 28, 40, 41, 47]
**Change in ACS**	4	High	453	MD R -1008.73 [-1664.75, -352.72]	0%; 0.80	65	[24–26, 47]
**Hospitalization**	3	Moderate	2348	RR F 0.78 [0.61, 0.99]	0%; 0.99	100	[24, 26, 34]
**All-cause mortality**	9	Moderate	3000	RR F 0.91 [0.79, 1.04]	0%; 0.44	81	[20, 23, 26, 34, 40, 41, 44, 45, 47]
**Cardiovascular mortality**	3	Moderate	2102	RR F 0.94 [0.76, 1.16]	0%; 0.80	84.5	[34, 44, 45]
**Hepercalcemia (**>**10.2 mg/dL)**	10	Moderate	957	RR F 0.43 [0.32, 0.56]	0%; 0.90	38	[20, 25, 26, 31, 41, 42, 44, 47–49]
**Hepercalcemia (**>**11.0 mg/dL)**	8	Moderate	605	RR F 0.22 [0.13, 0.37]	0%; 0.78	40	[20, 25, 30, 33, 39, 44, 45, 47]

Abbreviations: CACS, coronary artery calcification scores; ACS, aortic calcification scores

^a^Graduated by GRADE profiler

^b^Random-effects model

^c^Fixed-effects model

^d^Serum calcium-phosphate product

^e^Follow-up period (wk)

**Fig 7:**
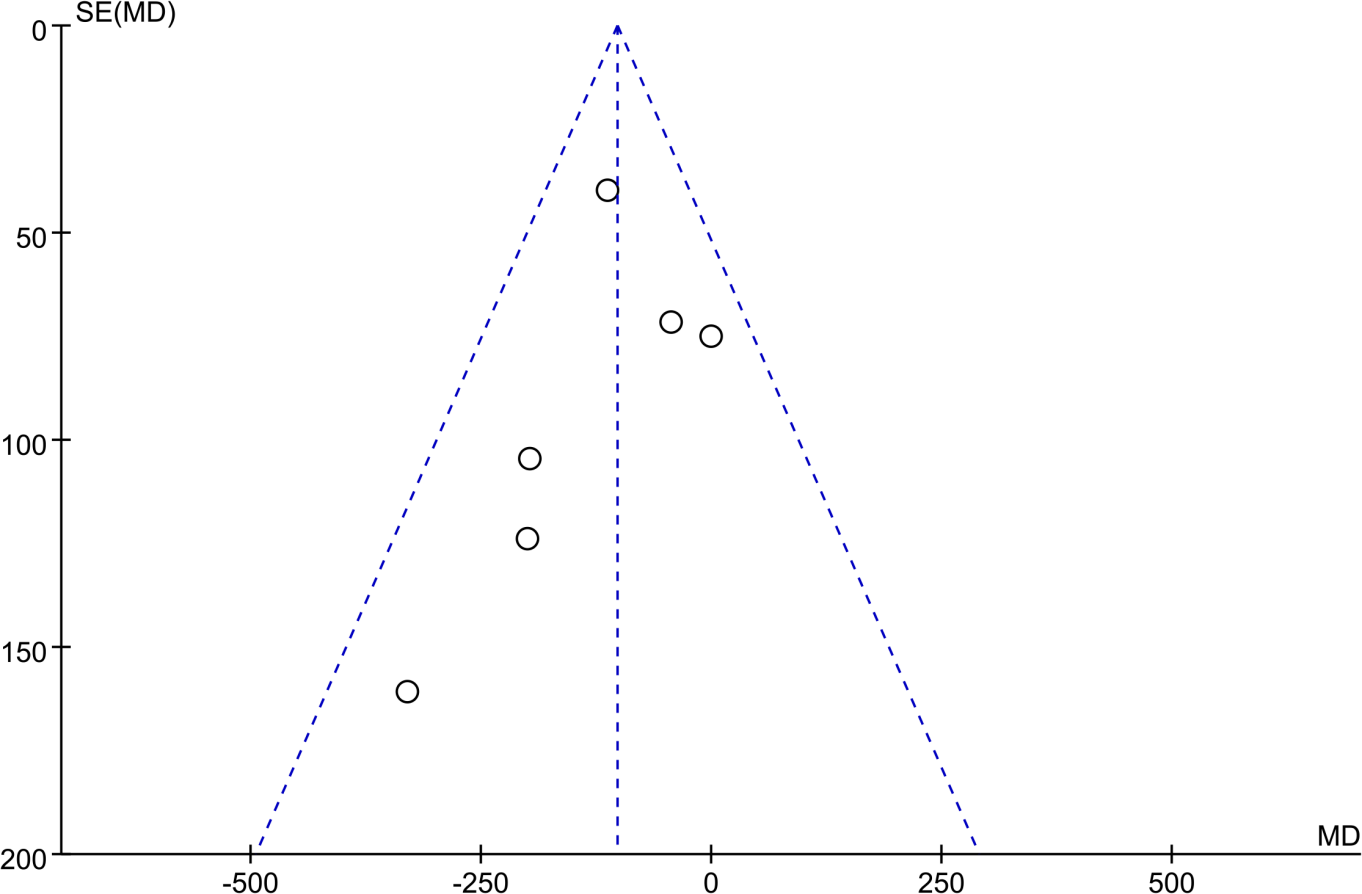
Funnel plot of the value of change of CACS.

Funnel plots revealed an approximately symmetrical distribution (Fig 7). Hence, publication bias was present

### Meta-Regression for Baseline Variables

As a result of high heterogeneity (I^2^ = 58%) in the analysis of phosphorous, we undertook a meta-regression using CMA and analyzed three factors: mean duration of dialysis; designed duration of the trial; and sample size of the trial. However, we did not find a significant factor (P>0.1) that contributed to heterogeneity (S6 Fig). Hence, an appropriate subgroup analysis was not carried out.

A meta-regression on CACS was performed using baseline variables: mean duration of dialysis; designed duration of the trial; and sample size of the trial. Partially because of the low heterogeneity (17%), we did not find a significant factor that contributed to the heterogeneity (S7 Fig and S8 Fig).

## Discussion

We carried out a meta-analysis to estimate the impact of sevelamer upon cardiovascular calcification, cardiovascular mortality, all-cause mortality, and hospitalization in patients on dialysis, and identified 31 studies (covering 23 trials with 4395 participants). Compared with CBPBs, sevelamer therapy resulted in smaller decreases in serum levels of phosphorus and a lower prevalence of hypercalcemia. A significant difference in the CACS and ACS was observed between sevelamer and CBPBs. Evidence that sevelamer reduced all-cause mortality or cardiovascular mortality was lacking. Also, there was a slight reduction in the duration of hospitalization with sevelamer therapy according to three RCTs.

Our review updates and complements the findings of earlier systematic reviews. It also includes >3000 additional participants, including a Dialysis Clinical Outcomes Revisited (DCOR) study [21] with 2103 participants—the largest randomized trial of sevelamer conducted.

Different to former meta-analyses, this meta-analysis found a significant difference in CACS and ACS. This phenomenon may be due to a better search strategy, as well as the inclusion of more trials and different types of patients. In the analysis of CACS, compared with eight RCTs on dialysis patients, a meta-analysis by Zhang 2010 [9] included four articles, and Jamal 2009 [10] included six trials in which a trial on predialysis patients was also evaluated. Similar to other reviews, CBPBs showed slightly better results for controlling serum levels of phosphate. In the analysis of serum levels of phosphate, we also undertook a meta-regression on serum levels of phosphate, and analyzed six factors but, unfortunately, factors that influenced the heterogeneity in serum levels of phosphate were found. We did not analyze the changes in sevelamer dose or CBPB dose in different treatment phases.

In this meta-analysis, we found a significant difference in CACS. Compared with CBPBs, sevelamer does not contain calcium, and is a type of non-calcium, non-magnesium, aluminum-free agent. As a result, sevelamer therapy can result in a smaller increase in serum levels of calcium and calcium-phosphate product. Also, the prevalence of hypercalcemia (defined as serum levels of calcium >10.2–10.5 mg/dL and serum levels of calcium >11.0 mg/dL) was also smaller. Serum levels of calcium are independent risk factors for vascular calcification, so less calcium in blood leads to a smaller increase in CACS for sevelamer therapy. However, our analysis showed no significant differences between sevelamer therapy and CBPB therapy in terms of cardiovascular mortality. A long time is required from vascular calcification to a cardiovascular event. Hence, sevelamer may reduce cardiovascular mortality in the long-term, and the fact that no significant evidence was observed for cardiovascular mortality may be due to short-term follow up.

Though sevelamer has less impact in controlling hyperphosphatemia, its use can result in a significant reduction in hospitalization. Moreover, a study showed that sevelamer-treated patients over 65 years old had a significant reduction hospitalization (P = 0.03) with a trend toward fewer hospital days (P = 0.08). In this respect, sevelamer can enhance the quality of life of patients.

Previous reviews showed no evidence to recommend use of sevelamer because there was no evidence to show that sevelamer has clinically meaningful benefits. However, our meta-analysis showed favorable use of sevelamer, especially for patients with hypercalcemia or high CACS. Also, compared with calcium-phosphate binders, the available trials mostly showed a clinically relevant beneficial effect of sevelamer.

The strengths of this meta-analysis were the number of participants and studies that we evaluated. Indeed, this is the largest systematic review of RCTs on dialysis patients to examine the effect of sevelamer compared with CBPB therapy on kidney-related serum measurements, CACS, ACS, hospitalization, and other endpoints of clinical safety.

However, several limitations must be considered. Unpublished reports could not be identified, which might have biased our results. Also, we could not assess the dosing schedules of sevelamer therapy and CBPB therapy (including dosing escalations and maximal dosing schemes), which may have contributed to the heterogeneity of our analysis (especially for the analysis of serum levels of phosphate). Patients undergoing hemodialysis or peritoneal dialysis were studied in the populations. With only four studies focusing on adequate allocation concealment, the quality of trials was not very high. Also, the duration of follow-up was short except for four Dialysis Clinical Outcomes Revisited trials. Intention-to-treat analysis was not used in some trials. In addition, some trials did not describe the number of dropouts.

In summary, compared with CBPBs, sevelamer has virtually no advantage in terms of the control of serum levels of phosphate, but it can decrease in the prevalence of hypercalcemia, and benefits vascular calcification in the long-term. We can conclude that sevelamer improves clinically relevant outcomes in ESRD patients on dialysis. Therefore, routine use of sevelamer in dialysis patients is recommended in patients that already have control of serum levels of phosphate, and if patients may suffer, or already are suffering, from hypercalcemia or cardiovascular disease. Those with severe hyperphosphatemia are recommended to choose CBPB therapy (at least in the short-term).

## Supporting Information

S1 Fig PRISMA 2009 Checklist. (PDF)

S2 Fig Risk of bias graph. (TIF)

S3 Fig Summary of risk of bias. (TIF)

S4 Fig Summary of findings tables. (TIF)

S5 Fig Linear regression of CACS and LDL. (TIF)

S6 Fig Meta-regression for baseline variables on serum phosphate. (TIF)

S7 Fig Meta-regression for baseline variables on the change of CACS. (TIF)

S8 Fig Regression graph of the change of CACS on total sample. (TIF)
