# Supplementary figures and images for: New Conclusions Regarding Comparison of Sevelamer and Calcium-Based Phosphate Binders in Coronary-Artery Calcification for Dialysis Patients: A Meta-Analysis of Randomized Controlled Trials
Source: PLoS One. 2015 Jul 31;10(7):e0133938. doi: 10.1371/journal.pone.0133938 (PMC4521824; doi:10.1371/journal.pone.0133938)

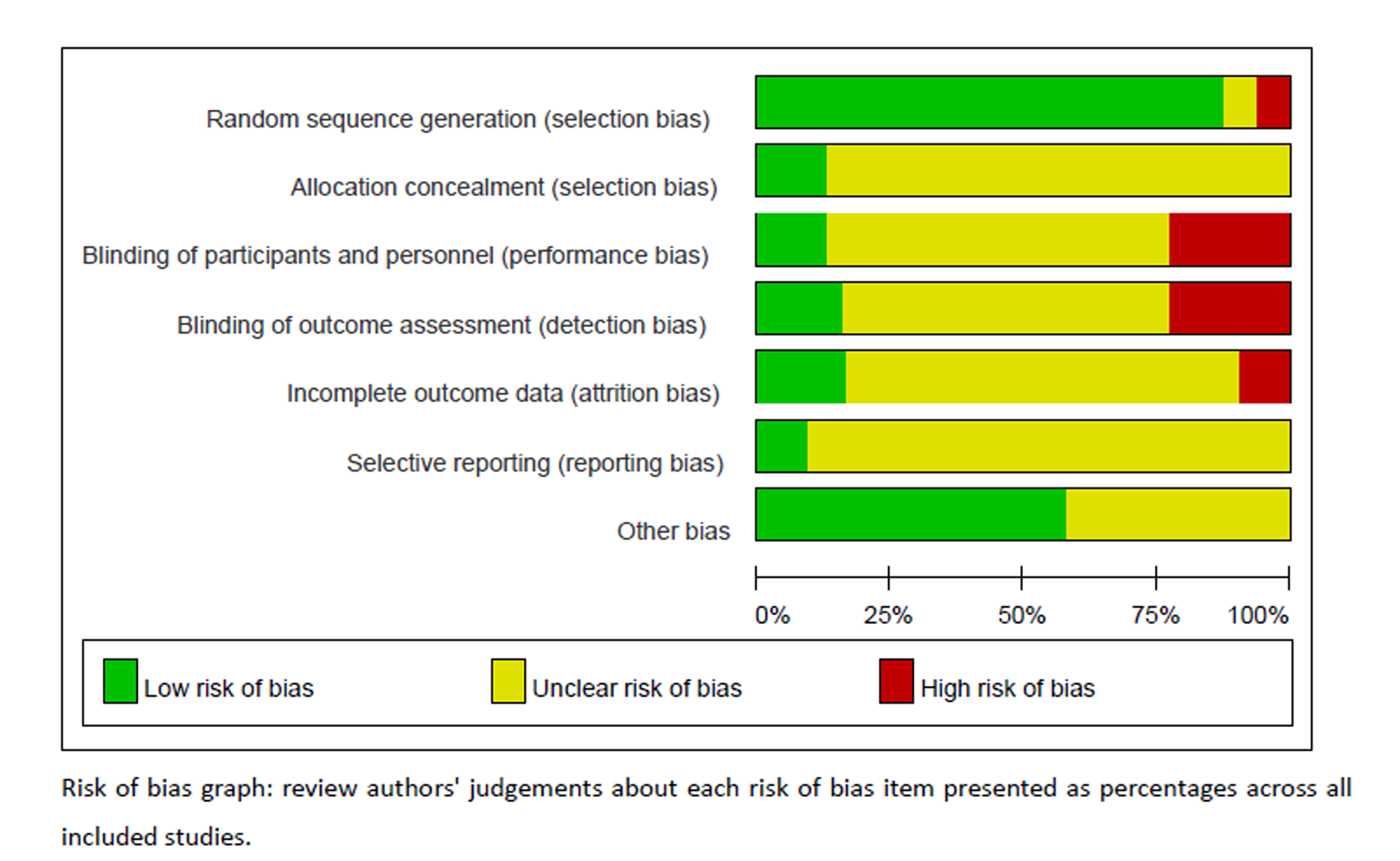

Supplement: S2 Fig — (TIF) [file pone.0133938.s002.tif]

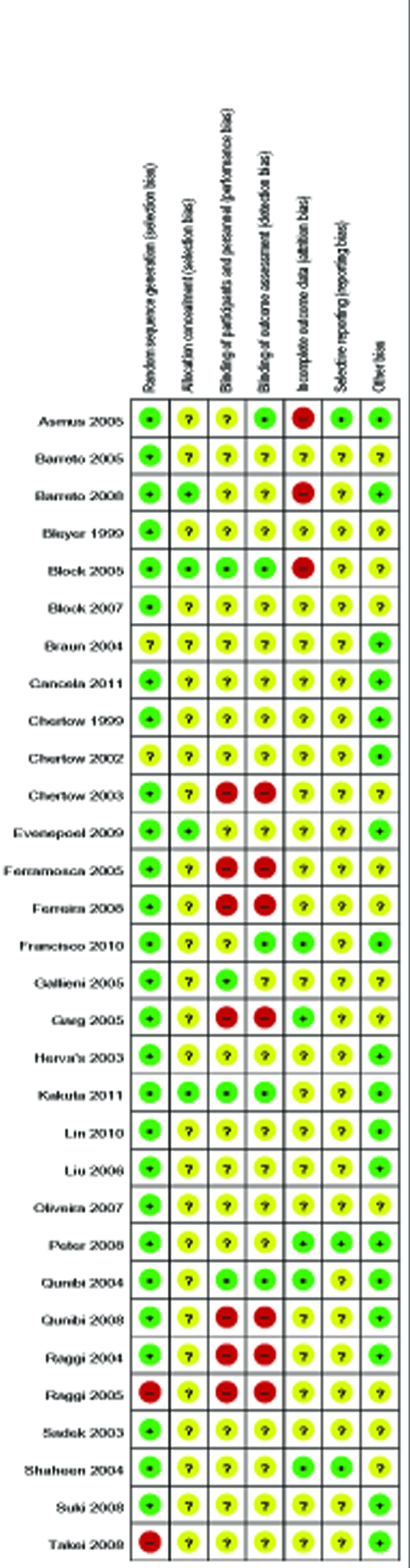

Supplement: S3 Fig — (TIF) [file pone.0133938.s003.tif]

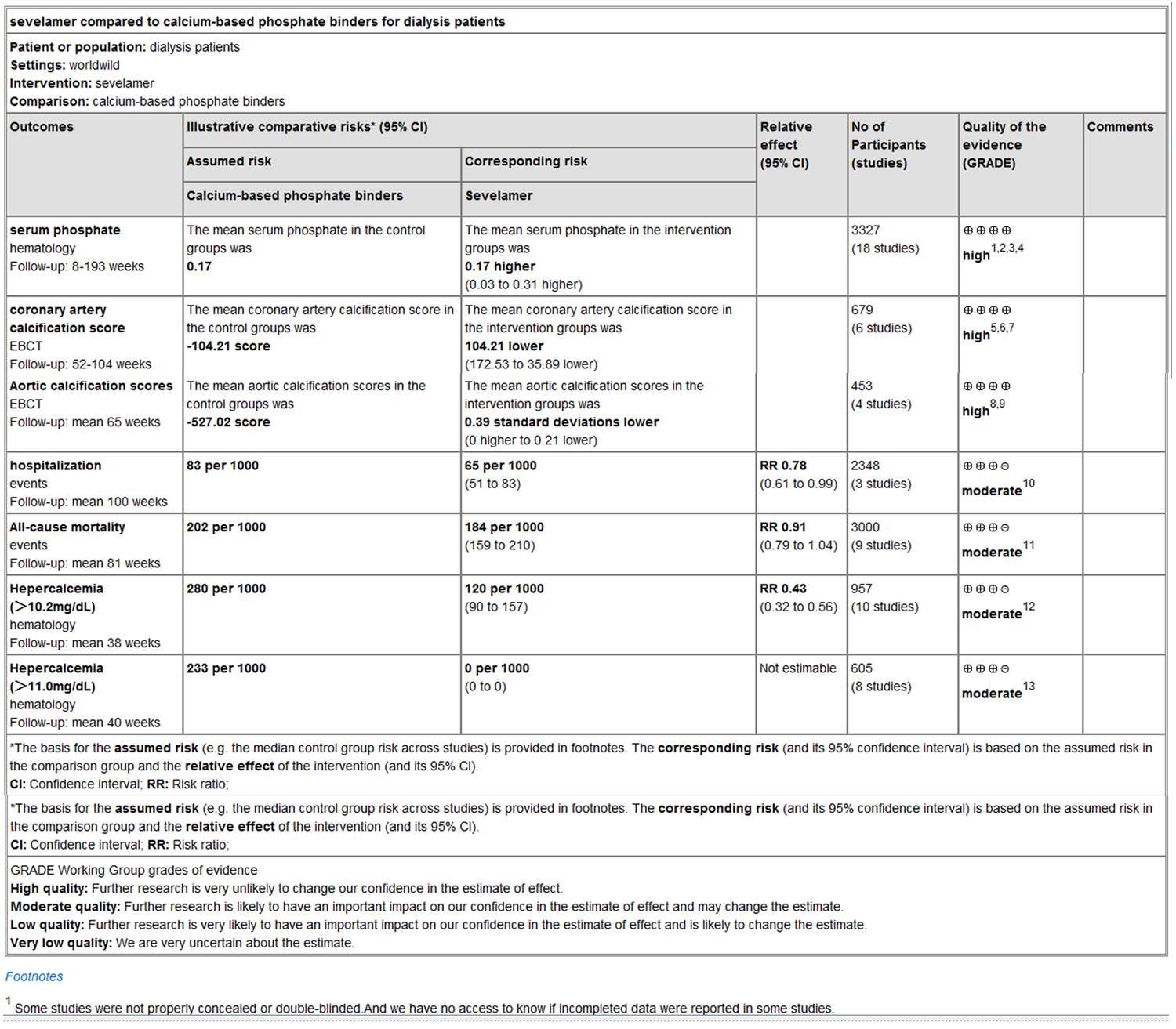

Supplement: S4 Fig — (TIF) [file pone.0133938.s004.tif]

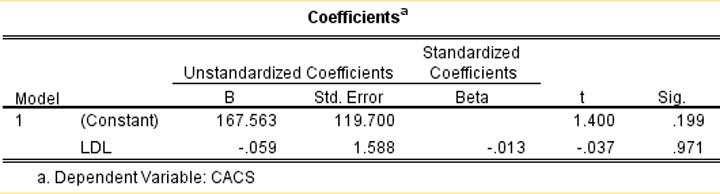

Supplement: S5 Fig — (TIF) [file pone.0133938.s005.tif]

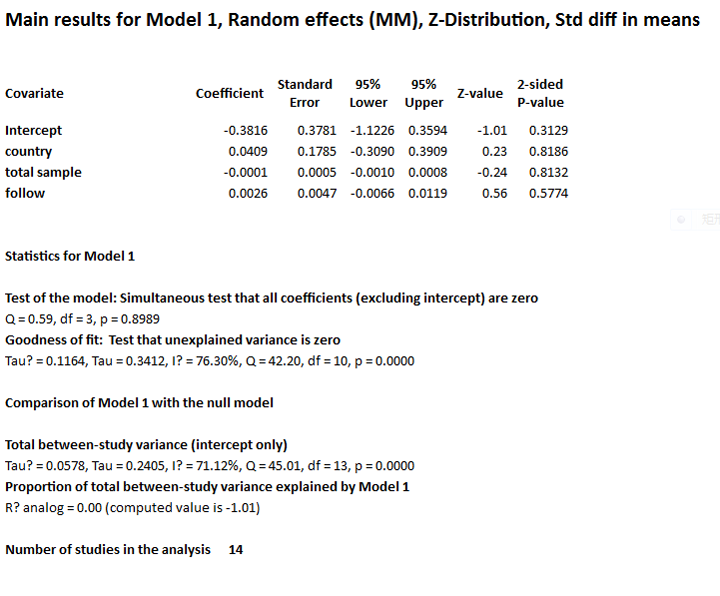

Supplement: S6 Fig — (TIF) [file pone.0133938.s006.tif]

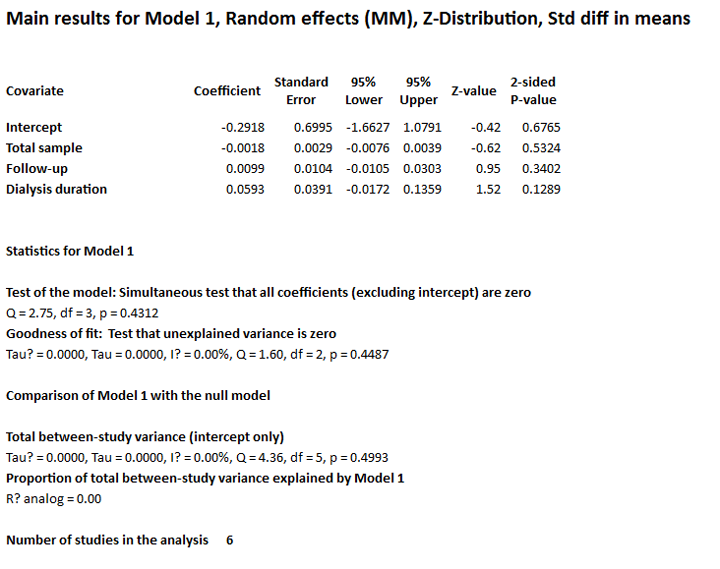

Supplement: S7 Fig — (TIF) [file pone.0133938.s007.tif]

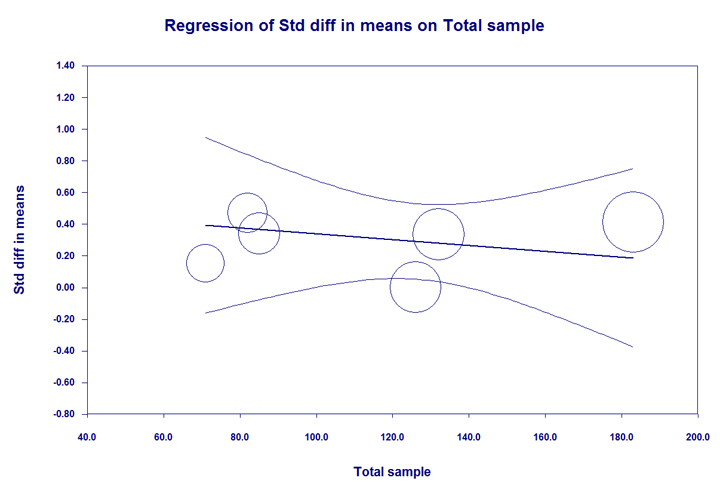

Supplement: S8 Fig — (TIF) [file pone.0133938.s008.tif]
